# Supplementary material for: Understanding Physical Activity Behavior in Ghanaian Adults with Type 2 Diabetes: A Qualitative Descriptive Study
Source: J Funct Morphol Kinesiol. 2023 Sep 5;8(3):127. doi: 10.3390/jfmk8030127 (PMC10532145; doi:10.3390/jfmk8030127)
Supplement: Supplementary file 1 [file jfmk-08-00127-s001.zip › jfmk-2539775-supplementary.pdf]

Supplementary file S1. Interview guide for consumers

Section A

1. Which adult group do you belong?
   a. 18-39 years ☐
   b. 40-54 years ☐
   c. 55-70 years ☐
   d. above 70 years ☐

2. What is your gender?
   a. Male ☐
   b. Female ☐
   c. Prefer not to disclose ☐

3. What is your marital status?
   a. Never married ☐
   b. Married ☐
   c. Cohabitating ☐
   d. Previously married ☐
   e. Prefer not to disclose ☐

4. What is your employment status?
   a. Employed ☐
   b. Not employed or retired ☐

5. Which part of Ghana do you live?
   a. Urban area (major cities like Accra, Kumasi, Tamale or district capital like Bekwai, Dodowa)
   b. Rural area (not within major cities or district capital)

6. What is your highest education level?
   a. No formal education/ Primary ☐
   b. Secondary ☐
   c. Tertiary or above ☐

7. Has your doctor or nurse informed you that you have type 2 diabetes?
   a. Yes ☐
   b. No ☐
8. If you answered 'Yes' to question 2, how long have you been diagnosed with type 2 diabetes? -
   ____

9. How active are you?
   a. Inactive ☐
   b. Moderately active ☐
   c. Highly active ☐

Section B

- Tell me more about the type of exercise you do on a weekly basis
- Tell me about your exercise goals
- What encourages you to exercise?
- Tell me more about what discourages you to exercise
- What can be done to support you to overcome exercise barriers?
- What things increase or decrease your self-confidence for being physically active?
- How do you know if you have achieved the amount of exercise you want to achieve?
